# Bacterial distribution and drug resistance in blood samples of children in Jiangxi Region, 2017–2021

**DOI:** 10.3389/fcimb.2023.1163312

**Published:** 2023-06-22

**Authors:** Yan Zhou, Shuping Zhou, Jun Peng, Liang Min, Qiang Chen, Jiangwei Ke

**Affiliations:** ^1^ Department of Clinical Laboratory, Jiangxi Provincial Children’s Hospital, Nanchang, Jiangxi, China; ^2^ Department of Clinical Laboratory, The Affiliated Children’s Hospital of Nanchang Medical College, Nanchang, Jiangxi, China

**Keywords:** children, bloodstream infection, blood culture, clinical isolate, drug resistance

## Abstract

**Objective:**

This study aims to investigate the distribution and drug resistance of bacteria in clinical blood culture specimens from children in Jiangxi province in recent years and to provide a foundation for preventing and treating bloodstream infection diseases in children.

**Methods:**

The study involved a statistical analysis of the isolation and drug resistance of bacterial strains obtained from blood culture specimens of children in Jiangxi province between 2017 and 2021. The analysis was performed using the WHONET 5.6 software.

**Results:**

A total of 7,977 bacterial strains were isolated from the blood samples of children between 2017 and 2021. Of these, 2,334 strains (29.3%) were identified as Gram-negative bacteria, and 5,643 strains (70.7%) were identified as Gram-positive bacteria. The most commonly isolated pathogens were coagulase-negative *Staphylococcus*, *Escherichia coli*, and *Staphylococcus aureus.* Among the Gram-negative bacteria, *Escherichia coli* (840 strains, 36.0%), *Klebsiella* pneumoniae (385 strains), *Salmonella* (283 strains), *Acinetobacter baumannii* (137 strains), and *Pseudomonas aeruginosa* (109 strains) were the most prevalent. Among the Gram-positive bacteria, coagulase-negative *Staphylococcus* (3,424 strains, 60.7%), *Staphylococcus aureus* (679 strains), *Streptococcus pneumoniae* (432 strains), *Enterococcus* sp. (292 strains), and *Streptococcus agalactiae* (192 strains) were the most common. Resistance to third-generation cephalosporins (cefotaxime/ceftriaxone) was observed in 45.9% and 56.0% of *Escherichia coli* and *Klebsiella pneumoniae* strains, respectively, while resistance to carbapenems was observed in 4.6% and 20.3% of these strains, respectively. Resistance to third-generation cephalosporins (cefotaxime/ceftriaxone) was observed in 15.5% of *Salmonella* strains, while resistance to imipenem was absent. Carbapenem resistance was observed in 17.1% (20/117) and 13% (14/108) of *Acinetobacter baumannii* and *Pseudomonas aeruginosa* strains, respectively. Methicillin-resistant *Staphylococcus aureus* (MRSA) was detected in 32.7% of the strains, while methicillin-resistant coagulase-negative *Staphylococcus* was detected in 64.3% of the coagulase-negative *Staphylococcus* strains. No *Staphylococcus* bacteria resistant to vancomycin were detected. Four strains of vancomycin-resistant *Enterococcus faecium* were detected over the 5-year period, and one strain of linezolid-resistant *Enterococcus faecalis* was detected.

**Conclusion:**

Gram-positive cocci were the most commonly isolated clinical pathogens in blood specimens from children in Jiangxi province. The composition of the pathogen species showed a slight change over the years. The detection ratios of pathogens varied with age group and season. Although the isolation rate of common carbapenem-resistant Enterobacter bacteria has decreased, it remains high. It is necessary to monitor the antimicrobial resistance of pathogens causing bloodstream infections in children more closely, and antimicrobial agents should be used with caution.

## 1 Introduction

Bloodstream infection (BSI) is a common infectious condition that is still associated with unacceptably high mortality and severe complications for many surviving patients (Cecconi et al., 2018; Rudd et al., 2020). In children, BSI carries a high disease burden, higher medical costs, and longer hospital stays, and it requires immediate and appropriate empirical antimicrobial treatment (Goudie et al., 2014). The distribution and drug resistance of bacteria cause BSI to vary in different regions, making it essential to follow the local data on the bacterial distribution and drug resistance for the initial selection of empirical antibiotics. Knowledge about the pathogen distribution causing pediatric BSI is crucial in identifying infection prevention strategies, tracking resistance patterns, and providing empirical antimicrobial therapy. Therefore, the primary objective of this study is to evaluate the spectrum of local microorganisms that cause BSI and their antimicrobial resistance (AMR) patterns in Jiangxi Province. We report on the distribution and drug resistance of pediatric BSI surveillance data obtained from 2017 to 2021 as part of the China Antimicrobial Resistance Surveillance System (CARSS) in 104 microbiological laboratories from 104 hospitals in Jiangxi Province, Southeast China. Dynamic monitoring of pathogenic bacteria distribution and drug resistance trends in pediatric BSI is of significant clinical importance.

## 2 Materials and methods

### 2.1 Strain source

This retrospective study aimed to investigate the distribution and drug resistance patterns of pathogens causing BSI in children in Jiangxi Province. Pathogens were isolated from blood culture samples obtained from children and collected from 104 microbiological laboratories as part of the Jiangxi Provincial Antimicrobial Resistance Surveillance System, a branch of the China Antimicrobial Resistance Surveillance System (CARSS). The participating hospitals included two secondary and three tertiary hospitals in Jiangxi Province, Southeast China. Every quarter, each laboratory member reported the bacterial identification and antibiotic sensitivity data to the CARSS. The study included only nonrepetitive strains isolated from the blood samples of patients.

### 2.2 Bacterial identification and antimicrobial susceptibility

The bacterial isolates were identified using a variety of methods, including the BD Phoenix automatic microbiological identification/drug sensitivity system (USA), the VITEK2-Compact automatic bacterial identification and drug sensitivity instrument, the Merier API system, and the Matrix-assisted laser desorption/ionization-time-of-flight mass spectrometry. Antimicrobial susceptibility testing was performed using the Kirby–Bauer method, the minimum inhibitory concentration (MIC) method, or the E-test method, and the results were interpreted by the breakpoint criteria recommended by the Clinical and Laboratory Standards Institute (CLSI) M100-S31 guidelines from 2021 (Spaulding et al., 2019). Microorganisms such as *Micrococcus* and *Bacillus* species were considered contaminants, and the status of coagulase-negative *Staphylococcus* (CoNS) as true pathogens or contaminants was confirmed in consultation with the attending clinicians.

### 2.3 Definitions

The study enrolled pediatric patients, defined as individuals aged less than 18 years old. The patients were further classified based on age as follows: newborns (less than 28 days old), infants (more than 28 days old and less than or equal to 1 year old), toddlers (more than 1 year old and less than or equal to 3 years old), preschoolers (more than 3 years old and less than or equal to 7 years old), school-aged children (more than 7 years old and less than or equal to 12 years old), and pubertal adolescents (more than 12 years old and less than or equal to 18 years old). Carbapenem-resistant Enterobacteriaceae (CRE) was defined as bacteria resistant to at least one carbapenem antibiotic, such as imipenem, meropenem, or ertapenem.

### 2.4 Data analysis

The data were analyzed using the WHONET software. Descriptive statistics, including numbers and percentages, were used to summarize the data. Changes in pathogen distribution by age and antimicrobial resistance over time were assessed using the chi-square test or Fisher’s exact test, as appropriate. The statistical analysis was performed using SPSS 17.0 software, and a *p*-value of less than 0.05 was considered statistically significant.

## 3 Results

### 3.1 Study population

Between 1 January 2017 and 31 December 2021, a total of 43,938 isolates from blood cultures were collected from patients admitted to hospitals in Jiangxi province. Among them, 7,977 isolates were consecutively collected from pediatric patients. **Table 1** presents the baseline characteristics of the study population. Of the pediatric patients, 4,776 (59.9%) were boys and 3,201 (40.1%) were girls, with an average age of 2.8 years. The majority of children were hospitalized in general pediatric wards (*n* = 3,236, 40.6%), followed by neonatal units (*n* = 2,390, 30.0%), pediatric surgery wards (*n* = 474, 5.9%), intensive care units (*n* = 416, 5.2%), and other wards (*n* = 1,461, 18.3%).

**Table 1:** Baseline patient characteristics.

Variable	Pediatric BSI (*N* = 7,977)
Sex (*n* (%))	
Males	4,776 (59.9%)
Females	3,201 (40.1%)
Average age (year)	2.8
Age categories (*n*/*N* (%))	
≤ 28 days	1,727 (21.6)
> 28 days, ≤ 1 year	3,430 (43.0)
> 1 year, ≤ 3 years	987 (12.4)
> 3 years, ≤ 7 years	720 (9.0)
> 7 years, ≤ 12 years	535 (6.7)
> 12 years, ≤ 18 years	578 (7.2)
Hospitalization unit (*n*/*N* (%))	
General pediatric wards	3,236 (40.6)
Neonatal units	2,390 (30.0)
Pediatric surgery wards	474 (5.9)
Intensive care unit	416 (5.2)
Others	1,461 (18.3)

### 3.2 Distribution of common pathogen pediatric BSI

From 2017 to 2021, 7,977 nonduplicate strains were isolated from pediatric blood cultures, consisting of 5,643 Gram-positive strains (70.7%) and 2,334 Gram-negative strains (29.3%). CoNS (3,424 strains, 42.9%) was the most common pathogen causing pediatric BSI, followed by *Escherichia coli* (840 strains, 10.5%), *Staphylococcus aureus* (679 strains, 8.5%), *Streptococcus pneumoniae* (432 strains, 5.4%), and *Klebsiella pneumoniae* (385 strains, 4.8%). Among the Gram-positive bacteria, CoNS accounted for 60.7% (3,424 strains), followed by *S. aureus* (679 strains), *S. pneumoniae* (432 strains), *Enterococcus* (292 strains), and *Streptococcus agalactiae* (192 strains). Among the Gram-negative bacteria, *E. coli* accounted for 36.0% (840 strains), followed by *K. pneumoniae* (385 strains), *Salmonella* (283 strains), *Acinetobacter baumannii* (137 strains), and *Pseudomonas aeruginosa* (109 strains). The top three pathogens remained unchanged from 2017 to 2021, namely CoNS, *E. coli*, and *S. aureus*. The results are presented in **Table 2** and **Table 3**.

**Table 2:** Distribution of pathogen isolates from blood culture (%).

Pathogen	Numbers	Pediatric BSI (%)
Gram-positive	**5,643**	**70.7**
*Coagulase-negative staphylococci*	3,424	60.7
*Staphylococcus aureus*	679	12.0
*Streptococcus pneumoniae*	432	7.7
*Streptococcus agalactiae*	192	3.4
*Enterococcus faecalis*	167	3.0
*Streptococcus viridans*, alpha-hem	111	2.0
Others	638	11.3
Gram-negative	**2,334**	**29.3**
*Escherichia coli*	840	36.0
*Klebsiella pneumoniae*	385	16.5
*Salmonella* sp.	283	12.1
*Acinetobacter baumannii*	137	5.9
*Pseudomonas aeruginosa*	109	4.7
*Stenotrophomonas maltophilia*	99	4.2
Others	481	20.6
**Total**	**7,977**	**100**

The principle of elimination and repetition: by eliminating the same patient and the same strain, only the first isolated bacteria are retained.

**Table 3:** The bacteria and constituent ratios of bacteria isolated from blood specimens, 2017–2021.

Pathogen	2017	2018	2019	2020	2021	Total	Pediatric BSI (%)
Coagulase-negative *staphylococci*	696	704	905	600	527	3,424	42.9
*Escherichia coli*	216	176	166	143	146	840	10.5
*Staphylococcus aureus*	145	138	144	135	123	679	8.5
*Streptococcus pneumoniae*	75	81	99	79	98	432	5.4
*Klebsiella pneumoniae*	82	69	82	66	87	385	4.8
*Salmonella* sp.	31	40	51	77	31	283	3.5
*Streptococcus agalactiae*	43	42	38	40	29	192	2.4
*Enterococcus faecalis*	34	46	39	18	84	167	2.1
*Acinetobacter baumannii*	34	36	28	17	22	137	1.7
*Streptococcus viridans*, *alpha-hem*	41	30	27	3	10	111	1.4
Others	275	276	319	220	233	1,327	16.6
Total	1,672	1,638	1,898	1,398	1,390	7,977	100

### 3.3 Distribution of pathogens in different age groups

**Figure 1:**
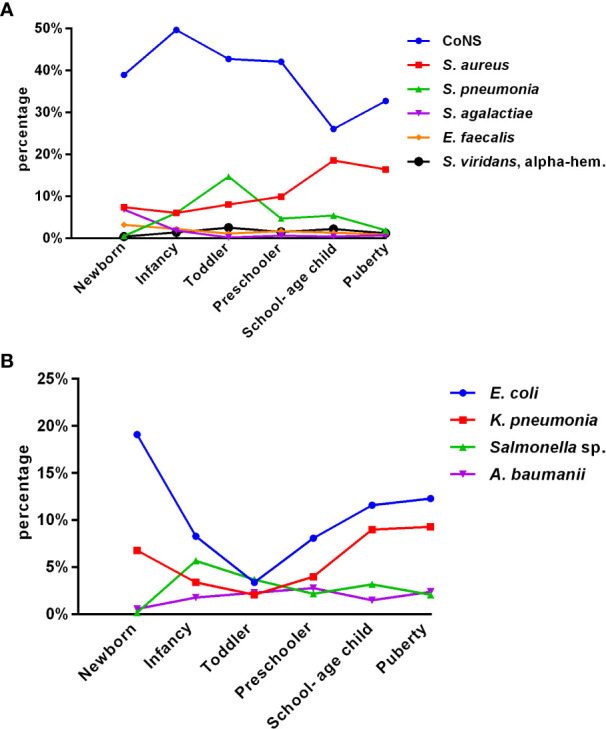
Distribution of the main pediatric BSI pathogens with age **(A**, **B)**. **(A)** Gram-positive bacteria (group 1). **(B)** Gram-negative bacteria (group 2).

Of the total isolates collected, 5,157 (64.6%) were from children aged ≤ 1 year, with 1,727 (21.6%) collected from newborns. **Figures 1A, B** depict the distribution of the main BSI pathogens among different age groups. CoNS was the predominant isolate in all pediatric age groups, with a gradual decrease in isolation rate as the age group increased, while that of *Staphylococcus aureus* increased.

Among all pediatric age groups, the neonatal group had the highest isolation rate of *Streptococcus agalactiae* (6.8%); as age increased, the rate gradually decreased. The rate of isolated *Enterococcus faecium* significantly declined after peaking at 3.2% in newborns. The rate of isolated *Streptococcus pneumoniae* peaked in toddlers and was significantly higher than that in puberty, except for newborns. Among Gram-negative bacteria, *Escherichia coli* and *Klebsiella pneumoniae* significantly decreased from newborn to toddler but increased from preschooler to puberty. The detection of *Salmonella* fluctuated greatly, with the lowest rate in newborns (0.2%) and the highest in infancy (5.7%), followed by a downward trend.

### 3.4 Common antimicrobial susceptibility changes of major Gram-positive pathogens over time

Gram-positive bacteria include CoNS, *Staphylococcus aureus*, *Streptococcus pneumoniae*, *Streptococcus agalactiae*, and *Enterococcus*. **Tables 4**, **5** presents the various degrees of antibiotic resistance exhibited by these bacteria. Staphylococci have a higher *incidence* of erythromycin resistance; *while* the detection rate of methicillin-resistant *Staphylococcus aureus* (MRSA) was 32.7%, methicillin-resistant coagulase-negative staphylococci (MRCNS) was 64.3%. No *Staphylococcus aureus* or coagulase-negative *staphylococci* were detected (**Table 5**). The resistance rate of *S. pneumoniae* to erythromycin and clindamycin was over 75%, and the resistance rate to penicillin G was consistently low, less than 5%. *Streptococcus agalactiae* showed 100% sensitivity to penicillin G and a certain resistance rate to levofloxacin, with an average resistance rate of 17.5%.

**Table 4:** The AMR fluctuations in major Gram-positive pathogens over time.

Pathogen	Antibiotic agent	Resistant rate					
		Pooled	2017	2018	2019	2020	2021
*S. aureus* (*n* = 679)	ERY	55.3	61.3	53.4	57.3	50.7	52.5
	OXA	32.3	28.4	29.5	27.7	19.7	19.7
	FOX	31.7	35.4	40.4	23.7	27.1	22.1
	CLI	27.4	34.4	30.3	28.5	21.7	21.7
	SXT	16.9	21.1	17.7	19.1	14.4	10.8
	RIF	5.0	5.3	4.9	6.7	4.9	2.6
	GEN	6.2	7.8	5.7	8.8	6.4	1.7
	LVX	6.4	12.8	6.8	2.7	4.8	5.3
	NIT	0	0	0	0	0	0
	LNZ	0	0	0	0	0	0
	VAN	0	0	0	0	0	0
CoNS (*n* = 3,424)	ERY	74.6	74.1	77.1	73.5	76.9	71.5
	OXA	69.0	66.2	68.5	69.6	67.2	63.1
	FOX	64.7	16.1	29.3	26.1	35.4	23.9
	CLI	37.0	41.3	42.9	39.7	32.2	25.5
	SXT	43.7	50.9	48.7	38.6	44.5	36.2
	RIF	7.7	9.2	8.5	7.8	6.5	6.0
	GEN	18.7	26.0	22.1	18.5	14.5	9.3
	LVX	18.2	17.6	18.4	16.5	19	20.4
	NIT	0.8	1.6	1.7	0	0	0
	LNZ	0.2	1.0	0	0	0	0.2
	VAN	0	0	0	0	0	0
*S. pneumonia* (*n* = 432)	ERY	93.4	94.4	86.1	94.9	96.1	93.9
	CLI	87.2	88.9	78.6	85.1	90.1	92.9
	SIT	57.4	61.1	51.3	52.1	57.5	65.2
	PEN	1.0	0	0	0	1.4	3.3
	LVX	0.7	0	2.5	0	0	1.0
	LNZ	0	0	0	0	0	0
	VAN	0	0	0	0	0	0
*S. agalactiae* (*n* = 192)	ERY	72.7	80.6	55.9	71.0	71.1	90.9
	CLI	58.7	67.5	50.0	55.6	53.8	70.8
	LVX	17.5	21.4	15.0	10.5	20.0	20.7
	PEN	0	0	0	0	0	0
	LNZ	0	0	0	0	0	0
	VAN	0	0	0	0	0	0
*E. faecalis* (*n* = 167)	AMP	21.7	29.0	31.8	25.6	5.9	3.2
	GEN	18.5	7.1	26.3	14.3	16.7	25.9
	RIF	54.2	55.6	42.1	68.8	55.6	54.5
	LVX	16.5	20.0	28.2	7.1	0	7.1
	LNZ	0.8	0	0	3.7	0	0
	VAN	0	0	0	0	0	0
	TEC	0	0	0	0	0	0
*E. faecium* (*n* = 93)	AMP	81.5	87.0	91.3	73.9	63.6	83.3
	GEH	21.1	27.3	22.7	21.7	9.1	16.7
	RIF	85.5	88.2	100	78.6	71.4	75.0
	LVX	70.3	85.7	100	50.0	60.0	60.0
	LNZ	0	0	0	0	0	0
	VAN	4.3	8.7	4.3	4.2	0	0
	TEC	1.5	6.7	0	0	0	0

ERY, erythromycin; OXA, oxacillin; FOX, cefoxitin; CLI, clindamycin; SXT, trimethoprim/sulfamethoxazole; RIF, rifampin; GEN, gentamicin; LVX, levofloxacin; NIT nitrofurantoin; LNZ, linezolid; VAN, vancomycin; PEN, penicillin G; AMP, ampicillin; GEH, gentamicin-high.

**Table 5:** Resistance of *Staphylococcus aureus* and coagulase-negative *Staphylococcus* to common antimicrobial agents.

Antibiotic agent	*S. aureus* (*n* = 679) MRSA (220/672, 32.7%)			CoNS (*n* = 3,424) MRCNS (2,146/3,340, 64.3%)		
	*N*	*R* (%)	*S* (%)	*N*	*R* (%)	*S* (%)
ERY	673	55.3	41.9	3,400	74.6	22.0
OXA	659	32.3	63.9	3,275	69.0	29.2
FOX	461	31.7	68.3	1,952	64.7	34.7
CLI	635	27.4	69.9	3,200	37.0	59.2
SXT	657	16.9	82.8	3,271	43.7	56.3
RIF	624	5.0	93.6	2,667	7.7	91.2
GEN	626	6.2	91.1	2,615	18.7	74.6
LVX	406	6.4	91.9	2,156	18.2	78.1
NIT	187	0	100	994	0.8	97.7
LNZ	650	0	100	3,115	0.2	99.8
VAN	672	0	97.3	3,310	0	97.4

ERY, erythromycin; OXA, oxacillin; FOX, cefoxitin; CLI, clindamycin; SXT, trimethoprim/sulfamethoxazole; RIF, rifampin; GEN, gentamicin; LVX, levofloxacin; NIT nitrofurantoin; LNZ, linezolid; TEC, teicoplanin; VAN, vancomycin.

### 3.5 Common antimicrobial susceptibility changes of major Gram-negative pathogens over time

**Figure 2:**
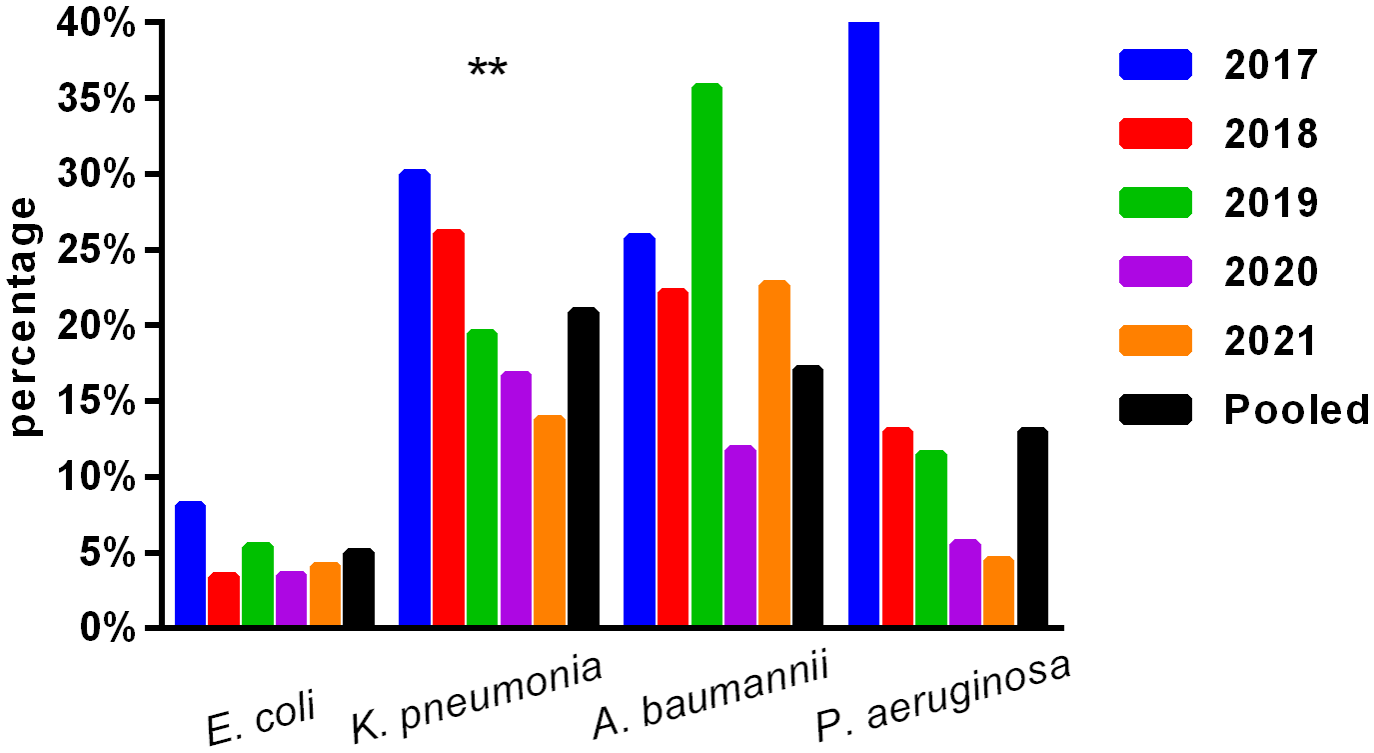
Isolation of carbapenem-resistant bacteria. ** , Asterisks indicate statistical significance as p-values <0.05.

The primary Gram-negative bacteria mainly include *Escherichia coli*, *Klebsiella pneumoniae*, *Salmonella* sp., *Acinetobacter baumannii*, and *Pseudomonas aeruginosa*. The overall resistance of *Klebsiella pneumoniae* was higher than that of *Escherichia coli*. The resistance rates of *Escherichia coli* and *Klebsiella pneumoniae* to third-generation cephalosporins (CTX/CRO) and imipenem were 45.9% and 56.0%, 4.6%, and 20.3%, respectively. In pediatric bloodstream infections, *Escherichia coli* exhibited high *in vitro* antibacterial activity against cefoperazone/sulbactam, piperacillin/tazobactam, amikacin, carbapenem, and polymyxin B, with a sensitivity rate exceeding 90%. The resistance rate to carbapenem declined from 8.1% in 2017 to 4.1% in 2021. *Klebsiella pneumoniae* demonstrated high *in vitro* antibacterial activity against carbapenem and amikacin, with a sensitivity rate exceeding 90%. The resistance rate to carbapenem decreased year by year, from 30.0% in 2017 to 13.8% in 2021. The resistance rates of *Salmonella* to cefotaxime and carbapenem were 14.3% and 2.5%, respectively. The results are presented in **Table 6** and **Figure 2**.

**Table 6:** The AMR fluctuations in major Gram-negative pathogens over time.

Pathogen	Antibiotic agent	Resistant rate					
		Pooled	2017	2018	2019	2020	2021
*E. coli* (*n* = 840)	AMP	82.8	83.6	82.9	86.3	82.4	78.9
	CZO	39.4	38	44.9	43.1	37.7	33.6
	CTX	46.5	44.9	47.8	50	44.4	47.2
	CAZ	22	24.7	21.4	24.2	20.1	20.1
	FEP	31.1	29.1	35.3	33.3	26.2	33.1
	ATM	25.6	26.1	22.3	27.1	29	25
	SAM	27	27.2	26.5	28.5	33.1	20.7
	AMC	20.5	16.8	21.8	27.8	25	15
	TZP	5.6	7.9	4.6	5.5	6.1	4.3
	SCF	4.6	3.8	3.4	3.8	6.4	5.8
	SXT	53.4	54.1	47.4	58.2	56.8	51.4
	GEN	29.8	33	32.4	31.8	24.4	23.9
	AK	0.7	0.9	0.6	0.6	0.7	0.7
	CIP	38.4	36	37.4	39	39.8	44.2
	LVX	33.6	30.2	31.8	37	34.1	39.2
	CHL	15.3	15.4	21.8	11.9	20.3	8.8
	IPM	4.6	5.7	3.6	5.7	3.9	4.3
	MEM	6	8.7	6.1	6.7	4	4
	POL	0	0	0	0	0	0
*K. pneumonia* (*n* = 719)	CZO	52.7	53.2	58.5	56.4	40.8	52.8
	CTX	59.8	67.4	61.8	61.4	54.5	45.5
	CAZ	44.2	50	41	43	35.5	50
	FEP	42.1	46.2	34.8	44.8	29.7	51.8
	ATM	42.7	42.9	30	39.3	38.9	58.5
	SAM	58.9	63.9	68.3	50.8	54.3	57.7
	AMC	38	52.1	52.9	45.5	36.4	16.9
	TZP	24.7	33.8	23.1	26	23.4	18.4
	SCF	22.2	23.5	26.9	24	19.6	21.7
	SXT	51.8	47.9	36.8	67.6	48.4	57
	GEN	22.5	24.7	14.5	21.6	34.6	19.4
	AK	7.6	9.9	8.7	8.5	4.6	5.7
	CIP	40.6	39	43.5	39.4	45.1	38.4
	LVX	21.9	19.5	21.2	27.1	19	22.2
	CHL	38.7	61.8	13.8	26.9	45.5	38.2
	IPM	20.3	28.8	23.5	20.3	16.9	14.1
	MEM	25.1	40	36.1	17.6	20	15.7
	POL	0	0	0	0	0	0
*Salmonella* sp. (*n* = 283)	AMP	46.5	53.3	38.5	61.5	42.9	43.3
	AMC	2.9	0	0	6.9	0	6.1
	SCF	0.6	0	5.6	0	0	0
	AMP	15.6	26.9	26.3	19.5	11.9	7.0
	TZP	3.4	10.7	13.2	0	1.4	0
	CAZ	10.3	10	12.8	6.0	8.5	13.6
	CTX	14.3	14.8	11.1	9.1	11.5	22.0
	FEP	11.1	23.1	15.8	2.2	7.5	13.2
	ATM	10.9	12.5	18.8	3.0	3.8	17.3
	IPM	0	0	0	0	0	0
	MEM	2.9	11.5	11.1	0	0	0
	CIP	14.7	20.7	25.0	15.6	10.3	9.7
	LVX	9.8	16.7	18.4	7.1	7.1	6.2
	SXT	31.8	29.0	22.5	32.6	38.7	30.5
	CHL	28.0	22.7	13.8	32.4	30.3	32.1
	TCY	35.8	30.0	17.4	75.0	35.6	34.0

AMP, ampicillin; CZO, cefazolin; CTX, cefotaxime; CAZ, ceftazidime; FEP, cefepime; ATM, aztreonam; SAM, ampicillin/sulbactam; AMC, amoxicillin/clavulanic acid; TZP, piperacillin/tazobactam; SCF, cefoperazone/sulbactam; SXT, trimethoprim/sulfamethoxazole; GEN, gentamicin; AMK, amikacin; CIP, ciprofloxacin; LVX, levofloxacin; CHL, chloramphenicol; IPM, imipenem; MEM, meropenem; POL, polymyxin B; TCY, tetracycline.

### 3.6 Seasonal distribution of main pediatric BSI pathogens

**Figure 3:**
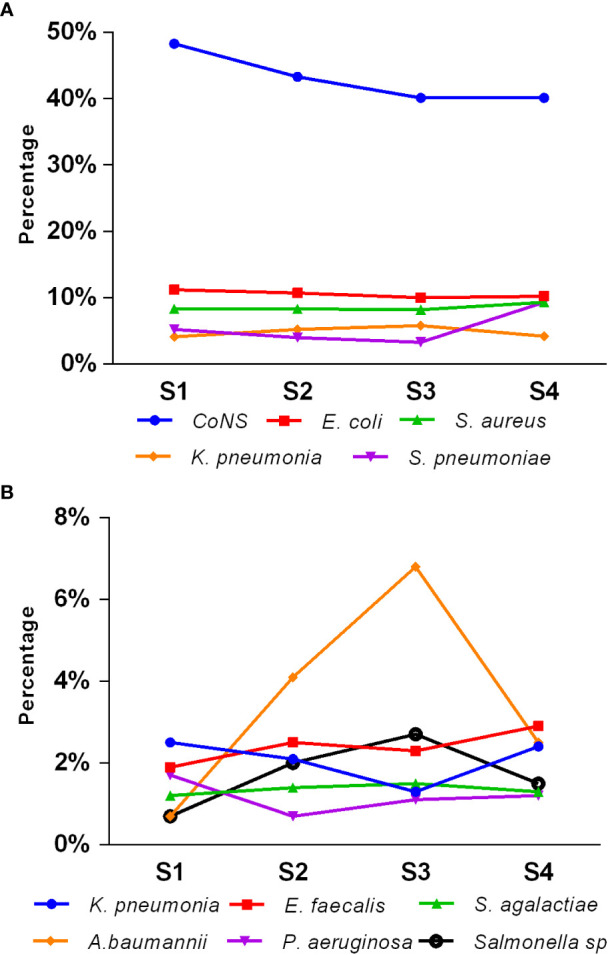
Seasonal distribution of main pediatric BSI pathogens **(A**, **B)**. S1, first quarter (spring); S2, second quarter (summer); S3, third quarter (autumn); S4, fourth quarter (winter).

**Figure 3** illustrates the seasonal distribution of the main pathogens causing pediatric BSIs. The overall isolation rate was higher in the second and third quarters (summer and autumn, 25.7% and 25.5%, respectively) compared to the fourth and first quarters (winter and spring, 24.3% and 24.6%, respectively) (*p* < 0.05). CoNS dominated across all seasons. The isolation rates of *E. coli*, *S. aureus*, *S. agalactiae*, and P. aeruginosa remained relatively stable throughout all four seasons. However, the isolation rates of CoNS, S. pneumoniae, *K. pneumoniae*, *Salmonella* species, *E. faecalis*, *E. faecium*, and *A. baumannii* showed statistically significant differences among the four quarters (*p* < 0.05). Notably, the isolation rate of *Streptococcus pneumoniae* was higher in the first and fourth quarters (winter and spring) than in the second and third quarters (summer and autumn). Conversely, *Salmonella* showed a higher isolation rate in the second and third quarters (summer and autumn) than in the first and fourth quarters (winter and spring).

## 4 Discussion

The incidence and mortality of sepsis have decreased globally; however, despite the decreasing age-standardized rates, sepsis continues to be a significant cause of health loss worldwide (Fleischmann-Struzek et al., 2018). Research data indicate that bloodstream infections are a leading cause of sepsis (Vincent et al., 2009; Hu et al., 2022), and overall, sepsis incidence peaks in early childhood, particularly in newborns and infancy. The high incidence of pediatric bloodstream infections is associated with prolonged hospital stays, higher healthcare costs, and increased patient mortality (Fleischmann-Struzek et al., 2018). Therefore, prompt and appropriate empirical antimicrobial treatment is essential. Adequate empirical antibiotic therapy is ideally associated with lower morbidity and mortality in various infectious syndromes. The literature highlights a growing emphasis on understanding local antimicrobial resistance patterns (AMR) and using regional antibiograms when selecting empirical therapy (Jones, 2001; Kajumbula et al., 2018).

Among the 7,977 pediatric patients in our study, 5,157 (64.6%) were children aged ≤ 1 year. Our findings indicate that pediatric BSI are highest in newborns and infants. This could be attributed to the immaturity of their immune systems and poor immune function, making them more susceptible to invasive infections. We observed that the constituent ratios of pathogens varied with age groups, and each age group had its own unique pathogen distribution characteristics. The isolation rate of CoNS decreased gradually with the increasing age group, whereas the isolation rate of *Staphylococcus aureus* increased. The isolation rate of *Streptococcus agalactiae* was highest (6.8%) in newborns but gradually decreased with increasing age. The isolation rate of Enterococcus *faecium* declined significantly after reaching its peak (3.2%) in newborns. The isolation rate of *Streptococcus pneumoniae* peaked in toddlers and was significantly higher than that in puberty, except for newborns. Among Gram-negative bacilli, the isolation rates of *Escherichia coli* and *Klebsiella pneumoniae* decreased significantly from newborns to toddlers but increased from preschoolers to puberty. The detection of *Salmonella* showed a fluctuating pattern, with the lowest rate in newborns (0.2%) and the highest in infancy (5.7%), followed by a downward trend. Understanding the distribution of pathogens in different age groups can guide physicians to prescribe more specific antibiotics in the future. The pathogen distribution in blood cultures varies widely. The drug resistance spectrum also differs, indicating that age is an essential factor in pathogen distribution and changes in resistance patterns over time. These results are consistent with those of a retrospective cohort study on the epidemiology of bloodstream infections in the USA from 2009 to 2016 (Spaulding et al., 2019).

In this study, the 5-year average distribution of pathogens was predominantly Gram-positive bacteria (70.7%). It is in line with the antimicrobial resistance surveillance of bacterial strains isolated from blood samples of children in 11 hospitals across China from 2016 to 2018, where Gram-positive bacteria were the leading pathogens isolated from blood culture samples (Jing et al., 2021). Consistent with previous studies conducted in various countries, CoNS was the most frequently isolated organism from blood cultures in hospitalized children (Huttunen et al., 2015; Larru et al., 2016; Zhang et al., 2022). However, the isolation of CoNS from a single blood culture can present a diagnostic challenge, as it may indicate colonization or contamination. The reported contamination rate in the literature is approximately 65.0% to 85.2% (Elzi et al., 2012; Karakullukçu et al., 2017). The distribution and drug resistance of bacteria cause BSI to vary by region. A systematic review and meta-analysis of bloodstream infections in children in low- and middle-income countries revealed that *Salmonella* is the most common pathogen causing bloodstream infections in Asia, followed by *Staphylococcus aureus*. While the primary pathogens in Africa are *Staphylococcus aureus*, *Streptococcus pneumoniae*, and *Escherichia coli* (Droz et al., 2019). In our study, the top five pathogens isolated from pediatric blood cultures were CoNS, *Escherichia coli*, *Staphylococcus aureus*, *Streptococcus pneumoniae*, and *Klebsiella pneumoniae*. Notably, the top three pathogens from 2017 to 2021 remained the same (**Tables 2**, **3**).

Over 5 years, this study found that the average detection rates of MRSA and MRCNS were 32.7% and 64.3%, respectively, and no vancomycin-resistant *Staphylococcus aureus* or coagulase-negative *staphylococci* were identified (**Table 5**). Notably, the isolation rate of MRSA decreased significantly in the study region (*p <*0.05), which differs from the remaining stable trends of MRSA prevalence observed in children in the Shandong Provincial Antimicrobial Resistance Surveillance System, a branch of the CARSS. Conversely, the study region’s MRCNS isolation rate remained stable (Wang et al., 2021). However, it should be noted that the detection rate of MRSA in this study was much higher than that observed in European countries. For instance, a significant decrease in the percentage of MRSA isolates was reported in the EU/EEA from 18.4% to 15.8% from 2017 to 2021 (Wang et al., 2023). Although the rate of MRSA is declining in European countries, the mortality rate of BSI caused by MRSA remains high (Dupper et al., 2019; Souli et al., 2019; Kimmig et al., 2020). Persistent *Staphylococcus aureus* bloodstream infection in pediatric patients has been associated with metastatic infection and septic shock, indicating the need for further investigation (Wang et al., 2023). *Streptococcus pneumoniae* is recognized as a major causative agent of community-acquired septicemia in children, with an incidence of 25% and a mortality rate of 8% (Asner et al., 2019). The monitoring data presented in this paper demonstrate that *Streptococcus pneumoniae* accounts for 5.4% of pediatric BSI cases, and the resistance rate of penicillin-resistant *Streptococcus pneumoniae* (PRSP) was 1.0%. *Streptococcus pneumoniae* retained penicillin hypersensitivity, indicating that penicillin, antibiotics containing beta-lactamase inhibitors, and third-generation cephalosporins remain effective treatments for *Streptococcus pneumoniae* BSI and can serve as a reference for clinical first-line treatment.

No significant change was observed in the resistance rates of Gram-positive bacteria to most antibiotics. Among Gram-negative bacteria, *Klebsiella pneumoniae* demonstrated a higher overall resistance compared to *Escherichia coli*, particularly to carbapenem, with resistance rates of both bacteria to ampicillin, first- and second-generation cephalosporins, cefotaxime, and compound sulfamethoxazole exceeding 30%. The resistance rates of *Escherichia coli* and *Klebsiella pneumoniae* to third-generation cephalosporins (CTX/CRO) and imipenem were 45.9% and 56.0%, 4.6%, and 20.3%, respectively. *Escherichia coli* isolated from pediatric BSI showed high *in vitro* antibacterial activity against cefoperazone/sulbactam, piperacillin/tazobactam, amikacin, carbapenem, and polymyxin B, with a sensitivity rate of more than 90%. The resistance rate to carbapenem decreased significantly year by year, from 8.1% in 2017 to 4.1% in 2021. Conversely, *Klebsiella pneumoniae* demonstrated high *in vitro* antibacterial activity against carbapenem and amikacin, with a sensitivity rate of more than 90%. The resistance rate to carbapenem significantly decreased from 30.0% in 2017 to 13.8% in 2021 (*p* < 0.05). Unfortunately, CRE have evolved from sporadic strains to globally prevalent drug-resistant strains over the last decade. The detection of CRE in children with bloodstream infections is not promising (Iovleva and Doi, 2017; Van Duin and Doi, 2017).

The present study investigated changes in CRE drug resistance isolated from children in China. The results indicated that the detection rate of CRE strains in children increased annually (6.4% overall), significantly higher than that of adult CRE strains. Moreover, the resistance of CRE to commonly used clinical antibiotics was severe (Guo et al., 2018). In this survey, the detection rate of CRE in children with bloodstream infections from 2017 to 2021 was found to be alarmingly high (9.1%), particularly carbapenem-resistant *Klebsiella pneumoniae* (CRKP), which reached 20.9%, far exceeding the surveillance data of children in China (Hu et al., 2016; Guo et al., 2018; Hu et al., 2018; Hu et al., 2020). These findings suggest that the drug resistance rate of Enterobacteriaceae bacteria causing invasive bloodstream infections in children may differ from that of non-invasive infections. Nevertheless, CRE has emerged as a critical cause of bloodstream infection control failure, treatment failure, and mortality in children, warranting greater attention (Nabarro et al., 2017). Resistance to amikacin was lower in *Escherichia coli* and *Klebsiella pneumoniae*, and the nonsensitive rate of polymyxin was lower (10%), which may be associated with limited clinical use in children. Amikacin may cause ototoxicity and hearing loss in children; however, when there is a clear indication for its use and no other viable alternatives, such drugs can still be considered under close monitoring for adverse reactions. *Salmonella* is one of the most important pathogens causing infant infectious diseases, leading to high morbidity and mortality rates and requiring hospitalization (Katiyo et al., 2019). Studies (Angelo et al., 2016) have revealed that newborns are susceptible to *salmonella* bacteremia, and 34% of blood culture isolates show resistance to first-line antibiotics (Angelo et al., 2016). In this survey, most of the patients with bloodstream infections caused by *Salmonella* were infants (> 28 days and ≤ 2 years old) (234/283, 82.7%), with newborns accounting for only 1.4% (4/283). The cefotaxime and carbapenem resistance rates to first-line antibiotics recommended for salmonella bacteremia were 14.3% and 2.5%, respectively, as shown in **Table 6** and **Figure 2**.

Previous studies have emphasized the importance of considering seasonal trends in healthcare-associated infections (Paul, 2012). Our survey demonstrated that the total pathogen isolation rate in children with bloodstream infections was higher during summer and autumn compared to winter and spring (*p* < 0.05). It suggests a potential association between pathogen distribution and the seasons. Specifically, we observed notable seasonal variations in certain bacteria. For instance, the isolation rate of *Salmonella* species was significantly higher in summer and autumn compared to winter and spring (*p* < 0.05). In contrast, the isolation rate of *Streptococcus pneumoniae* was significantly higher in winter and spring compared to summer and autumn (*p* < 0.05). These findings differ from previous studies (Guiraud and Post, 2017; Chamat-Hedemand et al., 2023). Further research is warranted to explore seasonal trends in pediatric bloodstream infections.

The limitations of this study were the inability to perform a detailed chart review for each episode and to characterize the clinical severity of each case of bacteremia. In addition, the rates of AMR may be overestimated because microbiology in this region tends to be a diagnostic tool within hospitals with more antibiotic pressure, and part of the samples were drawn from patients who were admitted for treatment failure and/or after receiving empirical antibiotics elsewhere. More strict studies and prospective trials are needed for broader coverage to avoid this potential bias.

## 5 Conclusion

Gram-positive cocci are the predominant clinical pathogens isolated from blood specimens in Jiangxi, with slight changes observed in the constituent species of these pathogens. The detection ratios of pathogens varied with age group and season. Although there has been a decrease in the isolation rate of common carbapenem-resistant Enterobacteriaceae, the rate remains high. Thus, there is a need to strengthen surveillance on the antimicrobial resistance of pathogens causing bloodstream infections in children and promote the rational use of antimicrobial agents.

## Data availability statement

The original contributions presented in the study are included in the article/supplementary material. Further inquiries can be directed to the corresponding authors.

## Ethics statement

The studies involving human participants were reviewed and approved by the Ethics Committee (IRB) of Jiangxi Provincial Children’s Hospital (the Affiliated Children’s Hospital of Nanchang Medical College). Written informed consent for participation was not required for this study in accordance with national legislation and institutional requirements. No potentially identifiable human images or data are presented in this study.

## Author contributions

JK and QC conceived and designed the experiments. SZ, JP, and LM analyzed the data. YZ wrote the manuscript. All authors read and approved the final manuscript. All authors contributed toward data analysis, drafting, and revising the manuscript and agreed to be accountable for all aspects of the work. All authors contributed to the article and approved the submitted version.
